# The Importance of Argan Oil in Medicine and Cosmetology

**DOI:** 10.3390/nu16203573

**Published:** 2024-10-21

**Authors:** Agata Serrafi, Fatima Chegdani, Faïza Bennis, Marta Kepinska

**Affiliations:** 1 Department of Immunochemistry and Chemistry, Wroclaw Medical University, ul. M. Skłodowskiej-Curie 48/50, 50-369 Wroclaw, Poland; agata.serrafi@umw.edu.pl; 2 Laboratory of Immunology and Biodiversity, Department of Biology, Faculty of Sciences Aïn Chock, Hassan II University of Casablanca, Route El Jadida, BP 5366 Maarif, Casablanca 20100, Morocco; fchegdani@yahoo.fr (F.C.); faizabennis@yahoo.fr (F.B.); 3 Department of Pharmaceutical Biochemistry, Faculty of Pharmacy, Wroclaw Medical University, Borowska 211a, 50-556 Wroclaw, Poland

**Keywords:** argan oil, argan tree, tocopherols, phenolic compounds

## Abstract

Argan oil, rich in unsaturated fatty acids and polyphenols, exerts beneficial effects on both the intestinal and skin microbiotas. In the gut, it promotes the growth of beneficial bacteria, such as *lactobacilli*, while reducing pathogenic bacteria, due to its anti-inflammatory properties that help maintain microbial balance. Additionally, it improves the integrity of the intestinal mucosa, reducing the risk of dysbiosis. On the skin, argan oil hydrates and balances the lipid environment, creating a favorable setting for beneficial microorganisms, while also possessing antimicrobial and anti-inflammatory properties that soothe conditions like eczema and acne. Thus, argan oil is valuable for overall health, supporting digestion and skin health. The objective of this review is to provide a summary of the benefits of argan oil for alternative and complementary medicine. An exhaustive search of the literature was carried out using targeted keywords. A set of 83 articles were selected and analyzed. As the mechanisms of action of argan oil are not completely understood, this work highlighted the benefits of this oil by analyzing its nutritional properties and its beneficial effects on the intestinal and skin microbiotas. Indeed, argan oil is valuable for overall health.

## 1. Introduction

*Argania spinosa*, also called iron argan or argan tree, belongs to the *Sapotaceae* family. It is prevalent in southwestern Morocco, around Essaouira, Agadir, Marrakech, Tiznit, and Taroudant and in the Negev Desert in Israel [1]. Argan forests protect the Earth’s surface from the effects of heavy rains and wind erosion. The argan tree adapts very well to difficult environmental conditions related to drought. In 1998, this species was included on the UNESCO (United Nations Educational, Scientific and Cultural Organization) list of protected biospheres [1,2]. The argan tree is a long-living, evergreen, thorny tree with a twisted, gnarled trunk, reaching a height of approximately 32.81 ft. It generally lives for about 150–200 years, although there are known cases of 400-year-old trees. It reaches full productivity only after fifty years. One tree can provide 1 L of oil per year. The fruits take one year to develop, ripening from June to July. They are yellow, resembling a plum, with flesh, a nut, and one to three fatty seeds inside. Black and dry fruits fall from trees [3,4]. Argan oil, extracted from the kernels of the argan fruit, is renowned for its high concentration of vital unsaturated fatty acids, such as oleic and linoleic acids, which have both health and nutritional advantages [5]. Beyond its oil, the argan tree plays a crucial role in sustainable development through the utilization of its byproducts. For example, the oil extraction process produces argan pulp and argan press cake (APC), which are used in a variety of applications. APC is a residue abundant in phenolic compounds, fatty acids, and proteins and is used in cosmetic formulations, animal feed supplements, and cell proliferation enhancement [6]. Argan pulp contains soluble sugars, dietary fibers, and phenolic chemicals, making it a flexible element in functional foods [5].

The bioactive compounds found in argan oil, such as polyphenols and fatty acids, influence immunity and gut metabolism [5]. They have antimicrobial activity against pathogenic gut microbiota, contributing to overall gut health. Moreover, these compounds have anti-inflammatory properties, as well as prebiotic qualities, promoting the growth of beneficial gut bacteria [6,7,8,9,10].

The intestinal microbiota, which is made up of a variety of commensal microorganisms such as yeasts, fungi, viruses, bacteria, and archaea, is intimately associated with the immune system and the digestive mucosa, and it is vital to human health [11]. Maintaining intestinal physiology and general health depends on this intricate ecology, which may change over time due to several factors such as antibiotic use, nutrition, and environment [12]. A wide range of illnesses, from metabolic disorders to mental health conditions, have been linked to dysbiosis, or imbalances in the gut microbiota [13]. Intestinal microbiota dysbiosis causes inflammation and can be treated with conventional therapies; however, side effects are frequent. Agents such as argan oil provide a potential natural alternative treatment by moderating this dysbiosis through their bioactive substances and anti-inflammatory and antioxidant properties.

Argan oil, called liquid gold, is one of the most popular oils in the world and the most expensive vegetable oil. The capacity of argan oil to regulate the composition of the intestinal microbiota indicates its therapeutic potential for gut health. Its rich polyphenol, essential fatty acid, and tocopherol profile contributes to a balanced microbiota, which in turn reduces inflammation of the intestinal mucosa, enhancing gut barrier integrity and promoting overall intestinal health. This suggests that argan oil may be useful in the management of inflammatory bowel disease (IBD) and other gut-related disorders. However, more investigation is necessary to completely understand the molecular mechanisms underlying the effects of argan oil on intestinal health and any clinical consequences, and this increased knowledge could help design innovative dietary treatments [5,7,8,9,10,11,12,13].

Argan oil can be used directly on the skin and combined with regularly used cosmetics, and the cosmetic market, both in Europe and globally, is already offering many cosmetics containing argan oil. Argan oil is increasingly appreciated and willingly used by consumers [11,13], and this increased interest is causing many manufacturers to use it in their cosmetic production; however, few disclose its percentage composition and confirmed effect on the skin.

Despite the potential benefits, the precise relationship among gut microbiota, argan oil, and host health remains inadequately understood. This review aims to clarify the biological importance of argan oil on host health and provide a summary of in vitro and in vivo evidence (animal and human models) of the effects of argan oil and the potential impact on gut microbiota and intestinal inflammation. The purpose of this article is also to discuss the chemical composition and the biological evaluation that has been carried out so far on argan oil.

## 2. Comparison of Argan Oil with Other Oils

**Figure 1:**
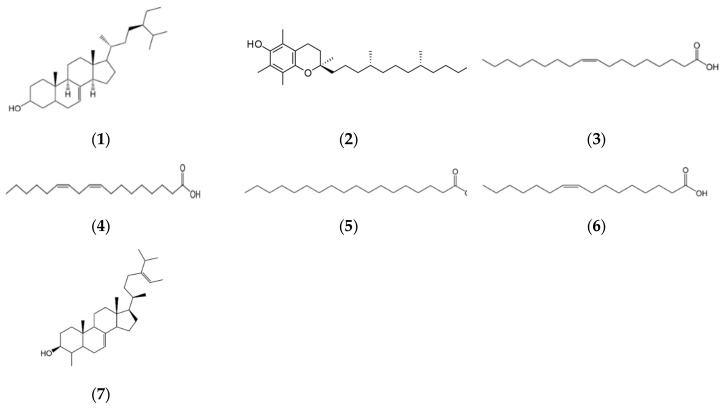
Examples of structures of fatty acids, sterols, tocopherols, and phenolic compounds of argan oil: (**1**) schottenol, (**2**) α-tocopherol, (**3**) oleic acid, (**4**) linoleic acid, (**5**) stearic acid, (**6**) palmitoleic acid, and (**7**) 4-α-methylstigmasta-7,24-28-diene-3β-ol.

Argan oil has a fatty acid composition similar to sesame and peanut oils but contains a smaller amount of oleic acid (about 45%) than erucic-free olive and rapeseed oils (about 60%), which are considered the healthiest. The stability of argan oil is comparable to sunflower oil; after heating it to 63.5 °C, no hydroperoxides were detected (Nuclear Magnetic Resonance (NMR)) as a result of oxygen absorption. Argan oil lacks β-sitosterol (δ-5 sterol) but does contain spinasterol (Spina) and schottenol (δ-7 sterols) (Figure 1). The level of α-tocopherol in argan oil (50 mg/kg) is less than in olive oil (approximately 190 mg/kg) and in sunflower oil (approximately 532 mg/kg). The squalene content in argan oil is approximately 3130 mg/kg, while in olive oil, it is 4990 mg/kg and in sunflower oil, it is only 60 mg/kg [14]. However, sunflower and olive oils contain higher amounts of phenolic compounds than argan oil [14,15]. Phenol derivatives present in argan oil include ferulic, caffeic, syringic, *p*-hydroxybenzoic, and vanillic acids; tyrosol; oleuropein; epicatechin; catechin; and catechol. Polyphenols are very valuable natural products that have antioxidant properties [15].

Due to the high price of argan oil, standardization methods are being sought that would confirm its identity and allow for the detection of adulteration with cheaper oils. In addition to testing the composition of fatty acids, gas chromatography can be used to determine the content of campesterol, which is found in trace amounts in argan oil, although there is a risk of adulteration with oil with partially removed campesterol [15]. Argan oil can also be obtained from seeds of fruits eaten by goats, in which case, it has different organoleptic properties related to the different composition of volatile fractions. The use of volatile compound analysis to determine the quality of argan oil requires further research [16].

## 3. Chemical Composition of Argan Oil and Its Derivatives

Argan oil is cold-pressed and then filtered. Due to a properly balanced composition of unsaturated fatty acids, argan oil has a light consistency, absorbs quickly, and reaches the deep layers of the skin. It is one of the strongest natural antioxidants and has become an important addition to anti-aging cosmetics [14].

### 3.1. Extraction Methods

**Figure 2:**
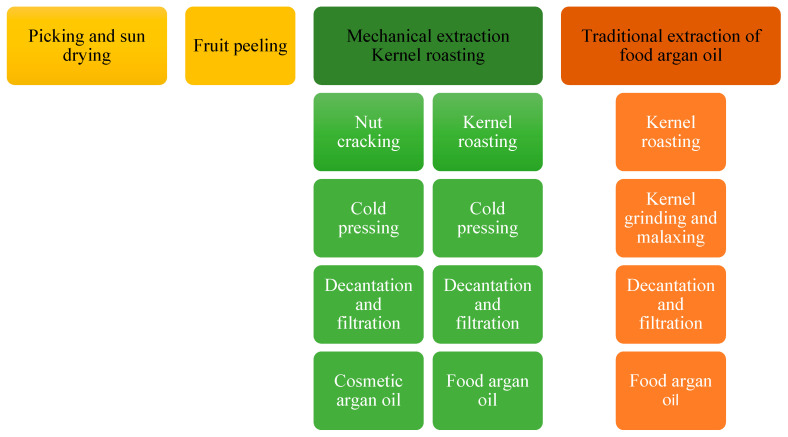
Scheme of argan oil extraction by the pressing method.

Argan oil, also called Moroccan gold or Berber almonds, has a yellow-brown color, a bitter nutty taste, and a slightly nutty scent. It is obtained from seeds [4] and can be obtained mechanically, using presses, which shortens the production process by 20% compared to the traditional method [14,15]. For industrial or laboratory purposes, argan oil can be obtained from crushed seeds through volatile lipophilic solvents. After evaporating the solvents and after one or two extraction cycles, an oil is obtained with a yield of 50% to 55%. This type of extraction allows one to obtain an oil that has unsatisfactory organoleptic properties but is still suitable for use in the cosmetic industry (Figure 2) [15].

The traditional method consists of very simple, hand-made operations, which are constantly being improved. The seeds extracted from the ripe fruit in their shells are crushed by smashing them with stones, and the crushed material is dried in the sun and gently roasted in clay pots. The roasted seeds are processed by mixing them by hand with warm water into a brown mass with the consistency of a thin dough, squeezed by hand and filtered. As a result, the oil in the form of a brown emulsion is decanted and then, after separating the emulsion, a clear, transparent layer of hazelnut-flavored oil is separated. The efficiency of this process is about 30%. The residue, in the form of a dark brown dough, still contains about 10% oil and is used as cattle feed. The oil is yellowish brown in color and contains variable water and unsaponifiable matter contents, as reported in the literature, and its quality is described as low [14]. The traditional method of obtaining it may also affect the composition and structure of triacylglycerols in the oil, but there are no data in the literature in this regard. Its durability is defined as lasting 14 days [14].

Another method of obtaining edible oil, which ensures better quality than the traditional method, is mechanical cold pressing in a press [14]. Probably in order to emphasize the difference in quality, this method is referred to in the literature as obtaining edible oil (although oil obtained traditionally is also used for food purposes). This oil is obtained from seeds roasted together with the surrounding sclerenchymatous tissue, then crushed and squeezed in presses. The beauty variety of the edible oil is pressed from unroasted seeds, as the authors write. Edible oil, as well as beauty oil, are produced mainly in Morocco [14,15].

For cosmetic purposes, argan oil is obtained by extraction with organic solvents such as cyclohexane or chloroform, mainly in Western Europe [4,15]. Argan oil for cosmetic purposes is characterized by a high content of unsaponifiable fraction, including antioxidants [15].

### 3.2. Fatty Acid Profile and Health Benefits

The chemical composition of most vegetable oils has a beneficial effect on the human body. Valuable properties of argan oil stem from its polyphenols, squalene, and tocopherols [15,16]. Glycerides constitute 99% of argan oil, of which 95% are triglycerides, comprised of oleic acid (43–49%), linoleic acid (29–36%), and trace amounts of linolenic acid. Oleic acid supports the transport of active ingredients naturally present in argan oil into the skin. There are also small amounts of saturated fatty acids in triglycerides. These acids are stearic (4–7%) and palmitic (11–15%). Unsaturated fatty acids are involved in several metabolic pathways, including chronic inflammation. Oleic acid is directly responsible for lowering blood pressure by regulating the structure of lipid membranes and inhibiting the activity of gelatinase A (MMP-2), an enzyme involved in the proliferation of cancer and Alzheimer’s disease [14,17]. Other organic compounds found in argan oil include polyphenols, sterols, carotenes, triterpene alcohols, and tocopherols. This is a group of anti-carcinogenic compounds. These compounds are part of the unsaponifiable fraction of argan oil, which constitutes only 1%. These ingredients are responsible for the stability of the oil and for its medicinal and cosmetic benefits (Table 1) [1,16].

### 3.3. Bioactive Compounds

Derivatives of phenols present in argan oil include ferulic, caffeic, syringic, *p*-hydroxybenzoic, and vanilla acids; tyrosol; oleuropein; epicatechin; catechin; and catechol [4]. Polyphenols are very valuable natural products that have antioxidant properties. They protect cells against damage caused by free radicals. They can also prevent atherosclerosis, cancer progression, and the development of pathogens. Oleuropein has anti-wrinkle and revitalizing properties [4,17,22].

Sterols present in argan oil constitute 20% of the unsaponifiable fraction. There are four sterols in argan oil: two major ones, spinasterol (44%) and dihydrospinasterol (shotenol, 48%), and two minor ones, avenasterol and 8,22-stigmastadien-3β-ol. These sterols have a regenerating effect on the epidermis [4]. Spina has anticancer properties, while shotenol has both anticancer and cytotoxic properties [4,23].

Triterpene alcohols constitute 20% of the unsaponifiable fraction of argan oil. Among the alcohols identified were tirukallol (27.9%), which relieves allergy symptoms, as well as butyrospermol (18.1%), β-amyrin (27.3%), lupeol (7.1%), and 24-methylcycloartanol (4.5%). Butyrospermol nourishes the skin both before and after sun exposure [1,15].

Carotenoids are represented by yellow xanthophylls that bind and transport oxygen. They can protect the skin against the harmful effects of solar radiation. They constitute 42% of the unsaponifiable fraction [3,4].

Argan oil is rich in tocopherol in the amount of 600–700 mg/kg. It is a strong antioxidant and the most efficient free radical scavenger. Argan oil contains 69% γ-tocopherol. It has been shown that γ-tocopherol is more effective than α-tocopherol in removing nitrogen-free radicals, as well as in preventing the proliferation of cancer cells. γ-tocopherol and its metabolite γ-carboxyethyl-hydroxychroman have an inhibitory effect on cyclooxygenase-2 isoenzyme (COX-2), which is activated during an inflammatory reaction. Inhibition of COX-2 leads to a reduction in symptoms of inflammation. Thanks to the presence of tocopherols, phenolic compounds, and carotenoids, argan oil protects the skin against the harmful effects of external factors and, above all, against free radicals, making it a potential ingredient in after-sun cosmetic products. It has a soothing effect on skin that is irritated and damaged by solar radiation. Vitamin E has a beneficial effect on the immune system. γ-tocopherol is also known for its role in the prevention of heart disease and prostate cancer [14,24].

The only hydrocarbon present in argan oil is squalene, and its content is approximately 0.3%. This compound helps remove xenobiotics from the body and lowers cholesterol levels. Studies have shown that squalene has protective properties against skin cancer [4].

There are differences in the chemical composition of argan oil, which depend on the geographical origin of the argan seeds as well as the methods of producing the oil. Argan oil obtained by cold pressing has the strongest antioxidant properties. Thermal treatment may reduce the anti-radical properties of argan oil obtained traditionally [25,26].

## 4. Biological Properties of Argan Oil

Research on argan oil has proven that its consumption has a beneficial effect on the prevention of cardiovascular diseases, inflammation, atherosclerosis, diabetes, hypertension, and cancer due to its cytotoxic, antiproliferative, and antioxidant activities. To date, there is one known case of an allergic reaction to argan oil, which occurred in a 34-year-old Moroccan man [6,24].

### 4.1. Antioxidant Activity

The phenolic compound-rich fraction of argan oil effectively prevents the oxidation of human low-density lipoproteins (LDLs). At concentrations ranging from 0 to 320 mg/mL, it significantly extended the lag phase, reduced the rate of lipid peroxidation (*p* < 0.01), diminished the formation of conjugated dienes and malonaldehyde (MDA), decreased vitamin E depletion, increased the phospholipid fluidity of high-density lipoproteins (HDLs) (*p* = 0.0004), and enhanced cholesterol efflux from macrophages in collaboration with HDLs [27]. Similar dose-dependent effects were observed with the pericarp extract, attributed to its ability to scavenge free radicals and chelate Cu(II) ions, which may lower the risk of early atherogenesis and prevent atherosclerosis and cardiovascular diseases. The pericarp extracts also exhibited cytotoxic effects (0–40 mg/mL) [28]. In vitro tests on ethyl acetate, petroleum ether extracts, and argan fruit decoction, containing polyphenols (89.4–218.0 mg/g as gallic acid), tannins (39.3–214.0 mg/g as catechin), flavonoids (3.4–11.1 mg/g as quercetin), and anthocyanins (0.74–10.92 mg/g as cyanidin), demonstrated antioxidant, antimalarial, and cytotoxic activities against human breast cancer cells (MCF7). The ethyl acetate extract and decoction showed good antioxidant activity, while the petroleum ether extract exhibited moderate activity, with 2,2-diphenyl-1-(2,4,6-trinitrophenyl)hydrazin-1-yl (DPPH) (IC50 = 32.3–600.8 μg/mL) and 2,2′-azino-bis(3-ethylbenzothiazoline-6-sulfonic acid (ABTS) (IC50 = 11.9–988.8 μg/mL) tests. The ethyl acetate extracts also demonstrated activity against chloroquine-resistant Plasmodium falciparum strains and cytotoxicity (IC_50_ = 42 to >100 μg/mL). A correlation was found between anthocyanin content and both antioxidant (R^2^ = 0.9867) and antimalarial (R^2^ = 0.8175) activities [29,30].

Inflammation is a universal response to various local tissue changes and is a key component of the acute-phase immune response, starting with a strong immune reaction aimed at eliminating harmful stimuli. This process triggers a cascade of chemical signals that activate leukocytes and lead to the production of inflammatory cytokines such as interleukin 1β (IL-1β), interleukin 6, and tumor necrosis factor-α (TNF-α) [31,32]. To prevent ongoing inflammation, it is essential to regulate this pro-inflammatory response and resolve inflammation to restore tissue homeostasis after the injury or pathogen has been cleared. While various anti-inflammatory drugs effectively reduce inflammation and severe pain by inhibiting pro-inflammatory signaling pathways, they often come with side effects [33,34]. Therefore, there is a growing need for natural products rich in antioxidants to mitigate these unwanted effects. Notably, argan oil has demonstrated therapeutic potential for cardiovascular disorders linked to obesity, hypertension, and insulin resistance. Prior research has confirmed its antioxidant, anti-inflammatory, anti-mutagenic, and anti-carcinogenic properties, along with its protective effects on neurological and liver health [35]. The rich composition of monounsaturated and saturated fatty acids, tocopherols, sterols, and polyphenols in argan oil enhances its therapeutic benefits, aiding in the management of hyperlipidemia, lipid homeostasis, oxidative stress, and inflammation, providing advantages for cardiovascular health and various dermatological conditions [19,36]. It also holds promise as a treatment for neuroinflammation [37].

Oxidative stress and inflammation are critical factors in neuroinflammation, where microglial dysfunction plays a significant role. Studies indicate that argan oil, particularly its phytosterols Schot and Spina, can reduce oxidative stress, inflammation, and peroxisome dysfunction in microglial cells, effectively lowering levels of reactive oxygen species (ROS) and nitric oxide (NO) and decreasing inflammatory markers such as IL-1β and TNF-α, thus improving peroxisomal function and providing protection against neuroinflammatory damage [38].

The dietary polyphenols in argan oil may also enhance gut microbiota, potentially offering antidepressant effects via the microbiota–gut–brain axis [37]. At nutritionally relevant doses, both argan oil and its unsaponifiable fraction exhibit significant anti-inflammatory effects comparable to those of diclofenac. These effects are primarily attributed to tocopherols and phytosterols, suggesting that argan oil may help alleviate the burden of inflammatory and degenerative diseases [9]. The phytosterols in argan oil can reduce the expression of pro-inflammatory cytokines like TNF-α and IL-1β induced by lipopolisacharyd (LPS) while promoting the expression of IL-4, an anti-inflammatory cytokine. Consequently, Schot and Spina display anti-inflammatory properties that may help protect against neurodegenerative diseases, providing strong evidence for the protective mechanisms of these key phytosterols in argan oil against LPS-induced neuroinflammation [38]. A study by Rozario Martínez et al. further highlighted that ethanolic extracts of argan pulp exhibited strong antioxidant activity in vitro, reducing inflammation and improving glycemic control, as evidenced by decreased inflammatory markers and increased antioxidant enzyme activity [5].

### 4.2. Effects of Argan Oil Consumption on Intestinal Microbiota Modulation

The intestinal microbiota, also known as the gut flora, is defined as a complex set of microorganisms called commensals, living inside the human digestive tract and, more specifically, in the gut [39,40]. This microbial community, non-pathogenic to humans, covers the surface of the intestinal mucosa. It is made up of fungi, viruses, yeasts, archaea, and, primarily, bacteria with a density of up to 10^14^ bacterial cells, 100-fold more than the cells of the human body [11].

The intestinal microbiota forms part of what is known as the intestinal ecosystem along with the digestive mucosa it covers and the immune system with which it interacts. It is considered an organ, living in symbiosis with the human organism and performing various functions [39].

The microbiota is complex and forms part of a dynamic intestinal ecosystem that constantly interacts with various bodily functions. Through its own role and functions, the microbiota itself helps to maintain a balance with its host [40]. The microbiota plays an essential role in intestinal physiology and human health and is increasingly being studied. A balanced and diverse intestinal microbiota is, in this sense, indicative of a good state of health [11,18].

The human microbiota plays a pivotal role in many bodily functions and overall health. The microbiota plays a significant role in digestive processes, particularly through the fermentation of dietary fibers into short-chain fatty acids (SCFAs). SCFAs are carboxylic acids with aliphatic tails of 1 to 6 carbons, of which acetate (C2), propionate (C3), and butyrate (C4) are the most abundant. Their production is carried out by bacteria such as the *Bifidobacterium* species (belonging to the actinobacteria phylum), which produce acetate and lactate during food digestion, and the main butyrate-producing bacteria in the human intestine belong to the Firmicutes phylum, in particular, *Faecalibacterium* [41]. These acids provide energy to colon cells and possess anti-inflammatory properties [42,43]. The gut microbiota could be used as a biomarker to aid in the diagnosis of disease [43]. Several alterations in gut microbiota are linked to different cancers, and modulation of metabolites produced by bacteria is able to regulate several brain cell functions [44]. Furthermore, it synthesizes vitamins and facilitates the absorption of essential nutrients [45]. The microbiota also modulates the immune system, allowing it to prevent autoimmune reactions and excessive inflammation [31], and it acts as a barrier against pathogens by occupying ecological niches and producing antimicrobial substances [46]. The composition and diversity of the microbiome are crucial for maintaining a symbiotic relationship with the host. It follows that an imbalance in the gut microbiota, or dysbiosis, has been linked to several diseases and conditions, including IBD, obesity, and diabetes, as well as cardiovascular disease [42,47,48]. This has the potential to influence nutrient absorption and fat storage [49]. Additionally, an imbalance in the gut microbiota has been linked to mental health disorders via the gut–brain axis, with alterations associated with anxiety, depression, and stress [50]. Moreover, the microbiota can influence the efficacy, toxicity, and therapeutic outcomes of drugs. By elucidating these interactions, it may be possible to develop personalized drug regimens that minimize adverse effects and maximize therapeutic outcomes [51]. This could be achieved by employing advances in metagenomics, metabolomics, nutrigenomics, and bioinformatics. These methods provide deeper insights into the complex interactions between the gut microbiota and the host [52].

The composition of the microbiota can change depending on many factors, such as the different stages of life, as it becomes more complex depending on the environment, diet, and hygiene, until around 3 years of age, when it becomes mature, stable, and identical in composition to the adult microbiota [53].

Regardless of age, argan oil is used by the Moroccans for various ailments. Argan oil taken orally has choleretic and lipolytic properties, as well as being a hepatoprotective and cholesterol-lowering agent. In addition, it has analgesic and anti-inflammatory properties. Argan oil is believed to strengthen the body and prevent miscarriages. The results of studies have shown that naturally occurring compounds, caffeic acid, *p*-coumaric acid and rutin, present in argan oil, can inhibit the growth and proliferation of bacteria, including *Staphylococcus aureus* and *Cutibacterium acnes* [29,54].

The modulation of the composition of the intestinal microbiota is associated with other factors, such as the mode of delivery and term of pregnancy, environment and geographical location, diet, and antibiotic intake [53]. Imbalances in its composition are responsible for disorders that play a role in causing and/or maintaining pathologies. Metabolic, immunological, cognitive, and psychiatric diseases may be a consequence of alterations in this flora and its functions [13]. Bacteria that form the dominant microbiota are divided into three main phyla: Firmicutes, Bacteroidetes, and Actinobacteria.

#### Probiotic Effects of Argan Oil

*Argania spinosa* is a source of various valuable extracts, each with distinct properties and applications. The most renowned extract is argan oil which is rich in several bioactive compounds that act as prebiotics [54,55]. Prebiotics are defined as non-digestible food components that stimulate the growth and/or activity of beneficial bacteria within the gut microbiota [56]. The enhancement of the growth of these beneficial microbes is, therefore, conducive to the establishment of a healthier gut microbiome environment [57,58]. A series of studies conducted on mice have demonstrated that argan oil supplementation can result in an increase in the abundance of beneficial bacteria, notably *Bifidobacterium* and *Lactobacillus*, while simultaneously reducing the prevalence of potentially harmful bacteria [54,59]. These changes contribute to the development of a more balanced microbial community, which is associated with an improvement in gut barrier function and a reduction in inflammation [60]. Furthermore, some preclinical studies have indicated that naturally derived dietary polyphenols can exert antidepressant-like properties by modulating the microbiota–gut–brain axis [37].

Another noteworthy extract is the APC. The observed beneficial effects are primarily attributable to the fermentation of dietary fibers by intestinal bacteria, leading to the production of SCFAs, such as acetate, propionate, and butyrate [43,61]. A recent study demonstrated that the consumption of dietary fibers similar to those present in the APC markedly enhanced the richness and diversity of beneficial gut bacterial, including *Lactobacillus* and *Bifidobacterium*, and facilitated the production of bacteria such as *Faecalibacterium prausnitzii* and *Roseburia* [61,62,63,64,65]. These changes are associated with improvements in gut health and a reduction in inflammation. The combination of bioactive compounds present in the APC, particularly polyphenols and fibers that act synergistically (e.g., gallic acid and catechin), possesses antimicrobial properties that can modulate the composition of the gut microbiota by reducing the growth of pathogenic bacteria, such as *Escherichia coli* and *Clostridium perfringens*, while simultaneously promoting the growth of beneficial bacteria [7,66,67,68,69,70].

The results of structure–activity relationship studies of phenolic acids found in argan oil show that some parameters, such as the basic chemical structure, position and number of hydroxyl groups, as well as their substituents in the phenolic ring and esterification of the carboxyl group, can affect antimicrobial activity. This compound can also disrupt the integrity of the cell membrane in Gram-positive and Gram-negative bacteria and change the charge, hydrophobicity, and permeability of the membrane surface [67]. Gallic acid can disrupt the permeability of the bacterial membrane and increase the accumulation of antibiotics in the microorganism [68,69,70].

Argan oil has been demonstrated to offer significant benefits for gut health through its anti-inflammatory and antioxidant properties [54]. The prebiotic properties of argan oil facilitate the formation of a balanced gut microbiome, thereby reducing inflammation in the intestinal mucosa and alleviating symptoms associated with IBD [71]. Furthermore, the antioxidant properties of argan oil mitigate oxidative stress, protecting against mucosal damage and maintaining the integrity of the gut barrier, which is essential in preventing inflammatory responses triggered by microbial dysbiosis [72,73]. The ability of argan oil to modulate the microbiota and reduce inflammation makes it a potential therapeutic agent for managing IBD [74]. Personalized treatment plans that incorporate argan oil could restore a healthy microbiota balance and improve disease outcomes. However, further research is necessary to elucidate the effects of argan oil on the gut microbiota and validate these findings in larger clinical trials, which would then pave the way for personalized dietary recommendations and therapeutic strategies.

### 4.3. Other Benefits of Argan Oil

Argan oil is known for a variety of additional benefits beyond its health properties. Moreover, saponins extracted from Argania spinosa at non-hemolytic concentrations were found to reduce erythrocyte hemolysis induced by free radicals by 53.2%, by 75% with 2 mM aspirin, and by 68% with acetaminophen. In contrast, vitamin E (0.3 μM) did not exhibit any antioxidant activity. The combined application of 1 mg/l saponin from A. spinosa and 0.3 μM vitamin E resulted in 68% protection against erythrocyte hemolysis, while the combination of acetaminophen (2 mM) and vitamin E (0.3 μM) did not demonstrate a synergistic effect. These findings confirm the antioxidant activity of saponins and their ability to enhance the antioxidant potential of vitamin E [28,30]. The initial in vivo studies conducted on hypertensive rats demonstrated that argan oil normalized blood pressure and induced hypocholesterolemia. This experiment was later replicated in gerbils (Meriones shawi) subjected to a high-calorie diet and physical inactivity, which resulted in hypertension, dyslipidemia, and hyperinsulinemia. After two months of administering argan oil at a dosage of 5 mg/kg/day, similar effects were observed in the rats: glycemia decreased by 4.4%, total cholesterol by 14.4%, LDLs by 32.5%, insulinemia by 26.8%, systolic blood pressure by 28.8%, and diastolic blood pressure by 30.5%. Additionally, there was a 27.9% increase in HDL levels and a 16.2% rise in triglycerides, while the animals’ body weight remained unaffected. The authors attributed these effects primarily to the unsaturated fatty acids in argan oil, alongside contributions from other components [75].

In another study, after five weeks of administering argan oil (10 mL/kg) to both hypertensive and normotensive rats, a significant reduction in systolic blood pressure (measured using the tail-cuff method, *p* < 0.05) and an increase (*p* < 0.01) in vascular endothelial response were noted in hypertensive rats. This response was characterized by the relaxation of the aortic ring and small mesenteric arteries induced by carbachol (10^−8^ to 10^−4^ M) after pre-contraction with phenylephrine. The use of the NO synthase inhibitor L-NW-nitroarginine (3 × 10^−5^ M) showed a significant contribution of NO to the observed relaxing effect. Furthermore, after blocking COX with indomethacin (10^−5^ M), the endothelial-dependent response included metabolites from the COX pathway. Argan oil significantly reduced (*p* < 0.05) the release of thromboxane A2 in the aorta and small mesenteric arteries of hypertensive rats, a finding corroborated by experiments utilizing a thromboxane A2 and prostaglandin H2 receptor antagonist, ICI 192,605 (10^−5^ M) [30]. The beneficial effects of argan oil may stem from its ability to decrease oxidative stress, as indicated by results from experiments involving antioxidants such as superoxide dismutase and catalase [76,77,78].

## 5. Experimental Models and Clinical Trials

In order to fully assess the anti-inflammatory and antioxidant effects of argan oil, numerous animal studies are needed. Some studies have explored the therapeutic potential of argan oil for oxidative stress and colonic inflammation in a rat model of ulcerative colitis (UC) induced by acetic acid [79]. Colitis was evaluated by body weight monitoring, stool consistency classification, tissue examination, and biochemical and hematological testing. The results revealed that argan oil effectively reduced inflammation and oxidative stress in rats with acetic acid-induced colitis. It also improved body weight, reduced colon damage, and alleviated symptoms such as bleeding and necrosis [80]. Argan oil was also shown to reduce markers of oxidative stress and enhance the activities of antioxidant enzymes. Although argan oil improved certain hematological and chemical serum parameters, it did not completely normalize all values. The presence of phenols and terpenoids in argan oil likely contributed to these protective effects. However, potential systemic hematological effects require further study to ensure safe medicinal use. Overall, argan oil has promising potential as a preventive or therapeutic agent for inflammatory bowel disorders [80].

The first in vivo studies conducted on rats with hypertension showed that argan oil caused a return to normal blood pressure and induced hypocholesterolemia. The test was repeated on gerbils (*Meriones shawi*) with an induced high-calorie diet and a lack of physical activity who exhibited hypertension, dyslipidemia, and hyperinsulinemia. After 2 months of administration of argan oil in the amount of 5 mg/kg/day, analogously to rats, glycemia decreased by 4.4%, total cholesterol by 14.4%, LDL fraction by 32.5%, insulinemia by 26.8%, systolic blood pressure by 28.8%, and diastolic blood pressure by 30.5%. An increase in HDL level by 27.9% and TG by 16.2% was also observed. The diet with argan oil did not affect the body weight of the animals. The authors attribute the dominant effect on the above activity to unsaturated fatty acids with the participation of other components of the oil [28]. After 5 weeks of administration of argan oil (10 mL/kg) to rats with hypertension and normotension, a reduction in systolic blood pressure (measured by the tail-cuff method) (*p* < 0.05) and an increase (*p* < 0.01) in the response of the vascular endothelium were observed in hypertensive rats, through relaxation of the aortic ring and small mesenteric arteries induced by cabochon (10^−8^–10^−4^ M), pre-constricted with phenylephrine. NO synthase inhibitor (*L-N-N-N*-arginine (3 × 10^−5^ M) caused a greater share of NO in the relaxing effect during the study. After blocking cyclooxygenase (COX) with indomethicin (10^−5^ M), the incorporation of COX pathway metabolites in the endothelium-dependent response was characterized.

Although animal studies are providing promising insights, clinical trials are still needed to validate these findings in human populations. Understanding the dose–response mechanisms and long-term effects of argan oil supplementation on the gut microbiota and clinical outcomes in IBD patients will be essential for its potential therapeutic application.

## 6. The Use of Argan Oil in the Cosmetic Industry

Argan oil with valuable nutritional and antioxidant values has found application in the food, cosmetic, and pharmaceutical industries. Natural ingredients present in the oil counteract signs of premature skin aging, have therapeutic significance in various skin and metabolic ailments, and reduce the risk of cardiovascular diseases, as well as cancer. Interest in argan oil is growing. Since argan oil is not widely used, research on its composition and effects on the human body is limited [1,14,26].

Thanks to its well-balanced composition of unsaturated fatty acids, argan oil has a light consistency, absorbs quickly, and penetrates deep into the skin. It is one of the strongest natural antioxidants and is highly sought after in anti-aging cosmetics [14]. Argan oil (INCI name: Argania spinosa kernel oil) offers numerous cosmetic benefits. It is easily absorbed, protects the epidermis from drying out, and moisturizes and smooths the skin. Coconut oil, which also contains polyphenols such as gallic, *p*-coumaric, and ferulic acids, as well as vitamin C and the B vitamins, is poorer in chemical composition and use in medicine than argan oil. On the other hand, olive oil is richer than argan oil in vitamin E (~3.6 mg) and also helps to treat damage caused by UV radiation. Although olive oil does not penetrate the hair strands like argan oil to nourish and repair them, it actually creates a natural protective barrier around damaged hair strands. Additionally, argan oil improves skin firmness and elasticity and revitalizes and supports cell renewal. Argan oil has a rejuvenating effect, preventing premature wrinkles, smoothing existing ones, and helping to prevent their recurrence. It also protects the skin from the harmful effects of free radicals. Suitable for sensitive, mature, and dry skin, argan oil is beneficial for people prone to allergies and acne.

The effect of argan oil on sebum secretion has also been studied. In a research study involving 2017 individuals with oily skin, the amount of sebum on the forehead and both cheeks was measured. Participants used a cream containing argan oil twice daily for four weeks. After this period, a reduction in sebum production and a visible improvement in the condition of oily facial skin were observed. However, continued use of the product did not lead to further improvements [22,24].

The improvement of the skin condition in the tested group is related to the chemical composition of argan oil, including glycerides and sterols. Oleic acid supports the transport of active ingredients naturally occurring in argan oil into the skin. Sterols present in argan oil constitute 20% of the unsaponifiable fraction. Argan oil contains four sterols: two main ones, i.e., spinasterol (44%) and dihydrospinasterol (schotenol, 48%), and two minor ones, avenasterol and 8,22-stigmastadien-3β-ol. These sterols have a regenerative effect on the epidermis.

The study involving a cream formulated with saw palmetto seed extracts, sesame seeds, and argan oil included 9 men and 11 women aged 17 to 50, with 16 participants having oily skin and 4 having combination skin. Conducted in winter, the experiment measured sebum levels on the forehead and both cheeks at the start and after 4 weeks using a Sebumeter device. An improvement in skin appearance was reported by 95% of the participants, with a 22% reduction in sebum levels and a 42% decrease in the area covered with oily spots, while the number of sebaceous glands remained unchanged [20]. Argan oil is also utilized for skin care before and after sun exposure, providing protection against harmful sunlight effects. It can be applied to sunburned skin as well [3,81,82]. Additionally, argan oil strengthens hair and protects it from sun damage [1,3,24]. It can also be massaged into nails, leading to increased resistance to breakage and greater hardness after just a few applications. Known as “Moroccan gold”, it enhances the nutritional content of the skin, hair, and nails [3,82]. In hairdressing salons, argan oil is used in its natural form as well as in various hair care products such as shampoos and masks. However, due to its cost, argan oil is sometimes substituted with cheaper alternatives. To ensure the quality of argan oil, analytical methods like gas chromatography have been developed [8,20,83].

## 7. Conclusions

Argan oil, often referred to as “liquid gold”, is one of the most popular and expensive vegetable oils in the world. Its ability to regulate the composition of the intestinal microbiota highlights its therapeutic potential for gut health. The rich profile of polyphenols, essential fatty acids, and tocopherols in argan oil contributes to a balanced microbiota, which helps reduce inflammation of the intestinal mucosa, thereby enhancing gut barrier integrity and promoting overall intestinal health. This suggests that argan oil may be beneficial in managing IBD and other gut-related disorders. However, further research is needed to fully understand the clinical implications and molecular mechanisms behind the effects of argan oil on intestinal health. Gaining insights into these connections could aid in the development of innovative dietary interventions and treatment plans aimed at improving gut health.

In addition to its health benefits, argan oil can be used directly on the skin and blended with cosmetics. The cosmetic market, both in Europe and globally, offers a wide range of products containing argan oil. The growing popularity of argan oil has led many manufacturers to incorporate it into their cosmetics, although few disclose its percentage composition or provide verified results. As a result, argan oil is increasingly appreciated and embraced by consumers. Argan oil could be a natural alternative for better overall health.

## Figures and Tables

**Table 1:** Examples of the chemical composition of various argan fractions and extracts.

Oil Fraction Compounds	Compounds		Traditional Extraction	Mechanical Press	References
Glyceridic fraction	Fatty acids (%)	Myristic acid (C14:0)	0.11 ± 0.01	≤0.2	TE [17] MP [18] SE [15,16] SFE [15]
		Palmitic acid (C16:0)	12.26 ± 0.19	13.25 ±1.75	
		Palmitoleic acid (C16:1)	0.03 ± 0.02	≤0.2	
		Stearic acid (C18:0)	5.55 ± 0.42	5.75 ± 1.45	
		Oleic acid (C18:1 *n*-9)	47.66 ± 0.56	46.05 ± 3.05	
		Linoleic acid (C18:2 *n*-6)	32.96 ± 0.76	32.65 ± 3.35	
		Linolenic acid (C18:3)	0.08 ± 0.01	—	
		Arachidic acid (C20:0)	0.38 ± 0.04	≤0.5	
		Behenic acid (C22:0)	0.12 ± 0.01	≤0.2	
	Monoacylglycerols (%)	Palmitoyl	4.95 ± 0.95	—	TE [19]
		Stearoyl	4 ± 0.5	—	
		Oleoyl and linoleoyl	81.8 ± 1.5	81.8 ± 1.5	
	Triacylglycerols		>4.14	≥19.0	TE [17] MP [20] SE [4]
Unsaponified fraction	Sterols (%)	Schottenol (Schot)	47.37 ± 0.77	46.5 ± 2.5	MP [17,20]
		Spina	35.80 ± 1.0	39 ± 5	
		Stigmasta-8,22-diene-3β-ol	4.53 ± 0.29	4.45 ± 1.25	
		Campesterol	0.2 ± 0.02	0.043	
	Triterpene alcohols (%)	Lupeol	—	7.1	MP [21]
		Butyrospermol	—	18.1	
		Tirucallol	—	27.9	
		β-amyrin	—	27.3	
		24-methylene cycloartenol	—	4.5	
		Citrostadienol	—		
	Tocopherols (mg/kg)	α-tocopherol	72 ± 10	59 ± 8	TE [21] MP [21] SFE [15]
		β-tocopherol	7 ± 2	6 ± 2	
		δ-tocopherol	82 ± 12	51 ± 8	
		γ-tocophero	585 ± 25	531 ± 25	
	Phenolic compounds (%)	Vanillic acid	12.38 ± 8.7	11.25 ± 6.4	TE [4] MP [4]
		Syringic acid	28.25 ± 6.66	25.48 ± 6.6	
		Ferulic acid	30.52 ± 22.4	31.49 ± 26.75	
		*p*-Hydroxybenzoic acid	10.86 ± 6.23	14.01 ± 7.11	

TE: Traditional extraction; MP: Mechanical press; SE: Solvent extraction; SFE: Supercritical fluid extraction.
